# Assessing Intertemporal Socioeconomic Inequalities in Alcohol Consumption in South Africa

**DOI:** 10.3389/fpubh.2021.606050

**Published:** 2021-05-11

**Authors:** Mayara Fontes Marx, Leslie London, Nadine Harker, John E. Ataguba

**Affiliations:** ^1^Health Economics Department, School of Public Health and Family Medicine, Health Science Faculty, University of Cape Town, Cape Town, South Africa; ^2^Health and Human Rights Programme, School of Public Health and Family Medicine, Health Science Faculty, University of Cape Town, Cape Town, South Africa; ^3^Alcohol Tobacco and Other Drug Research Unit, South African Medical Research Council, Cape Town, South Africa

**Keywords:** inequality, alcohol consumption, health equity, concentration index, socioeconomic inequality

## Abstract

**Background:** This paper assesses changes in the socioeconomic inequality in alcohol consumption by exploring whether alcohol consumption (current and binge drinkers) is more prevalent among the wealthier (pro-rich) or poorer (pro-poor) group over time.

**Methods:** Data come from the 2008, 2010/11, 2012, and 2014/15 waves of the National Income Dynamics Study (NIDS). Various equity stratifiers (sex, age, race, and rural/urban) are used to analyze the prevalence of alcohol consumption and to investigate differences in socioeconomic inequalities. Changes in socioeconomic inequality in alcohol consumption between 2008 and 2014/15 were also assessed using the concentration index.

**Results:** Current drinkers were more concentrated among richer South Africans, while binge drinkers were concentrated among the poorer population. For current drinkers, irrespective of sex, race, age, and urban, socioeconomic inequality in alcohol consumption had become less pro-rich between 2008 and 2014/15; while inequality in binge drinking, outside of the Asian/Indian and rural categories, had become less pro-poor between 2008 and 2014/15.

**Conclusion:** The results show evidence that binge drinking is a bigger problem among those of low-SES, young individuals, male and African populations. This paper concludes that the SA government should continue to push forward policies aiming to reduce the prevalence of binge drinking.

## Introduction

Alcohol use remains one of the biggest contributors to the risk of mortality worldwide. According to the World Health Organization (1), harmful alcohol use was responsible for 5.3% of deaths and 5.1% of the burden of disease and injury, equivalent to 132.6 million disability-adjusted life years (DALY, defined by WHO as the time lost due to premature death and the time lost due to time lived in less than full health). South Africa (SA) is particularly affected as one in 10 deaths are associated with alcohol use (2, 3). Previous studies using nationally representative survey data found that approximately half of men and one-fifth of women consume alcohol in SA. Of those who consume alcohol in SA, 48% of men and 32% of women binge drink (4). In 2015, the total per capita alcohol consumption in SA was 11.5 L of pure alcohol, and alcohol consumption per drinker was 27 L of pure alcohol—one of the highest levels of alcohol consumption in the world (5).

In addition to the direct toxic effects of alcohol products, studies suggest that the risk of disease and injury from alcohol consumption increases as individuals increase their alcohol intake. The idea is that the more you drink, your likelihood of being exposed to, or participating in risky behavior increases (6, 7). For instance, Taylor et al. (7) found in a meta-analysis that, for motor vehicle accidents and non-motor vehicle injury, the odds ratio increases by 1.24 and 1.30 per 10 g of alcohol consumption, respectively. In another meta-analysis that looked at the risk of adverse events such as HIV infection, alcohol consumption was found to increase the relative risk of HIV infection by 98%. In addition, the risk of HIV infection doubles for binge drinkers compared to non-binge drinkers (8).

The World Health Organization (9) stresses the need to examine inequities behind alcohol-related harms to understand the individuals and households that are more exposed and vulnerable to alcohol harms. There are a variety of factors at the individual/household level (for example age, education, and income) and the societal level (for example taxation policy, ease of alcohol availability, and norms around alcohol consumption) that influence alcohol consumption and alcohol related-harm (1, 10). Dahlgren and Whitehead (11) recognize that people are born with pre-existing characteristics and are subject to their communities' norms. They have different childhood experiences, education, and employment and housing opportunities (social determinants). These factors, involving interactions between individual characteristics and social determinants, experienced through the full lifespan, increase people's exposure and vulnerability to health hazards, including alcohol use (11). The inequalities in the distribution of these determinants are responsible for health inequalities between genders, communities, and societies (11). Socioeconomic status (SES) remains one of the most important social determinants of alcohol-attributable harm (1, 12, 13).

A systematic review looking at the relationship between the social determinants, inequities and alcohol use in Australia found that with the same alcohol consumption levels, individuals from low SES households experience more harmful effects of alcohol consumption than those from richer households (10). Moreover, a United Kingdom (UK) study using a population-based survey found that low SES individuals are more likely to be involved in high-risk drinking, which suggests a double disadvantage for alcohol-related harms—low SES individuals experience more harm for a given level of alcohol consumption and are more likely to drink at risky levels (14). In SA, a cross-sectional analysis using population-based survey found that low SES individuals are more likely to practice lifetime abstinence (never used alcohol), while high SES individuals are more likely to be current drinkers. For risky drinking, middle SES individuals are more likely to be binge drinkers, followed by low SES and high SES individuals (2). Alcohol consumption patterns are different for different alcoholic beverages. Wines and spirits are consumed mainly by high SES individuals, while beer is consumed mainly by low SES individuals (15, 16). These results might suggest that alcohol abuse in SA is not a reflection of genuine differences in alcohol consumption patterns among SES but may be a result of a more complex social problem.

This paper assesses changes in socioeconomic inequality in alcohol consumption by exploring whether alcohol consumption is concentrated among the wealthier group (pro-rich) or the poorer group (pro-poor). It also assesses whether these socioeconomic inequalities have changed over time. Based on current research, apart from an attempt to decompose socioeconomic inequality in alcohol consumption for men living in South African's informal settlements (17), this paper represents the first national analysis of changes in socioeconomic inequality in alcohol consumption in SA. It assesses this across various equity stratifiers [sex, age, race, and geographical location (rural vs. urban)] using datasets that span more than 5 years. A previous study assessing socioeconomic inequality (17) found that alcohol consumption is more concentrated among men of lower SES, but was confined to examining drinking patterns only in men in informal settlements at one point in time. While it is crucial to examine inequality in alcohol consumption in informal settlements in SA, it does not provide a broad picture of the entire country.

In this regard, this paper extends the analysis to the entire country using a nationally representative dataset and focusing on adults that consume alcohol and are more likely to binge drink. The analysis in this paper will assist in tracking SA's alcohol consumption patterns and socioeconomic inequality in alcohol consumption. This will assist in detecting early changes in alcohol risk behaviors such as an increase in binge drinking pattern by various equity stratifiers.

## Methods

### Data

This paper uses data from the National Income Dynamics Study (NIDS). The NIDS is a longitudinal study in SA that follows the same households over time (Wave 1, 2008; Wave 2, 2010–2011; Wave 3, 2012; and Wave 4, 2014–2015). It uses a face-to-face data collection process. New members are added to each survey wave by joining the households of continuing sample members (CSMs). NIDS contains household and adult surveys that can be used to produce cross-sectional and longitudinal estimates for a wide variety of adult and family well-being indicators at the micro-level. The Southern Africa Labor and Development Research Unit (SALDRU) based at the School of Economics, University of Cape Town manages the NIDS. The analysis in this paper uses the alcohol consumption data for the four NIDS waves in cross-section (18–21). While it is interesting to follow the same household over time, this paper adopted a different approach by looking at the entire population and how the distribution of alcohol consumption has changed in the entire population and across the selected equity stratifiers over time.

The NIDS data have a nationally representative sample of 7,296 households and 16,871 individuals in 2008; 9,127 households and 21,880 individuals in 2010/11; 10,219 households and 22,466 individuals in 2012; and 11,895 households and 26,819 individuals in 2014/15. The response rates were 94.9% for wave 1, 82.3% for wave 2, 81.1% for wave 3, 64.7% wave 4, and 86.5% for wave 5. The latest NIDS data—Wave-5, collected in 2017, does not contain alcohol data and will not be included in this analysis.

### Key Variables and Estimation Strategy

Table 1 summarizes the key variables used in the analysis. Two binary alcohol consumption variables were constructed: current drinker (yes/no) and binge drinking among drinkers—sometimes referred to as binge drinkers in this paper (yes/no). The variables were constructed using the NIDS adults survey questions: “how often do you drink alcohol?” and “on a day that you have an alcoholic drink, how many standard drinks do you usually have (a standard drink is a small glass of wine; a 330 ml can of regular beer, a tot of spirits, or a mixed drink).”

**Table 1:** Description of key variables.

**Variables**	**Definition**
Current Drinkers^*^	“1” if an adult consumes any amount of alcohol
	“0” otherwise
Binge Drinkers Among Drinkers^*^	“1” if an adult is a current alcohol drinker consuming 5 or more standard drinks on a single occasion for females^**^ and males
	“0” otherwise
Total household consumption expenditure (per capita)	Total household expenditure on food and non-food items (includes total food expenditure; total non-food expenditure; rental expenditure and imputed rent for owner-occupied housing) divided by the household size.

* *Amongst individuals 15 years and older*.

** *;Although the international literature suggests the use of four standard drinks as the benchmark for females, this was not possible due to the way the NIDS alcohol data are collected (that is the answers were grouped “1 or 2 standard drinks,” “3 or 4 standard drinks,” “5–6 standard drinks”)*.

This paper uses household consumption expenditure to assess the socioeconomic status of households. In developing countries, household consumption expenditure is a preferable measure of living standards than income. That is because income may be saved, and many households may not report actual income for many reasons including multiple sources of income (22) or for fear of taxation, among other reasons (23). Although using household consumption expenditure may underestimate the living standards of households with savings, this is not problematic as the interest is in current consumption.

All data cleaning, exploration and analysis was conducted using Stata 12 statistical software (24).

### Assessing Socioeconomic Inequalities in Alcohol Consumption and Other Key Variables

The concentration index (CI) was used in this analysis to assess socioeconomic inequalities in alcohol consumption (current and binge drinkers) in SA. The CI is a well-known and widely used index to assess socioeconomic inequality in health outcomes and indicators (22). The CI is derived from the concentration curve. Its values can vary from −1.0 (where all current drinking or binge drinking is concentrated in the poorest households) to +1.0 (where all current drinking or binge drinking is concentrated in the richest households).

The concentration indexes for alcohol consumption (current drinkers and binge drinkers) that measure the extent to which alcohol consumption is concentrated among the wealthier (pro-rich) or poorer (pro-poor) group, were calculated using the “convenient regression” approach to control for other variables (for example gender, race, age, and urban) in addition to SES (25). The “convenient regression” was performed in Stata 12 (24) to compute the concentration index (CI) using the following equation:

(1) $$2\sigma_{r}^{2}\left(\frac{h_{i}}{\mu }\right)\;=\;\alpha \;+\beta r+\;\gamma z\;+\;\varepsilon_{i}$$

where σ^2^ is the variance of the fractional rank (*r*) of household per capita consumption expenditure (SES), **z** is the vector of control variables, and the Ordinary Least Squares estimate, β, is the CI. Analyses conducted with adjustment for a binary outcome variable did not yield results that were materially different.

In addition to the CI for alcohol consumption, the Distributive Analysis Stata Package (DASP) (26) was used to assess the concentration indexes of various equity stratifying variables (sex, age groups, rural, and urban) among current and binge drinkers. This was used to assess, for example, whether female binge drinkers are more prevalent among poorer or wealthier groups. The DASP was also run using Stata 12 to obtain the CI as follows:

(2) $$CI=1-\frac{\hat{\xi }}{\hat{\mu }}$$

where $\hat{\xi }=\;\sum_{i=1}^{n}[\frac{{\left(V_{i}\right)}^{2}-\;{\left(V_{i+1}\right)}^{2}}{{\left(V_{1}\right)}^{2}}]h_{i};V_{i}=\sum_{j=i}^{n}w_{j}$ (the summation of sampling weights, *w*_*j*_) is such that the vector of total household consumption expenditure (SES), **x**, is arranged from the richest (*x*_1_) to the poorest individual or household (*x*_*n*_). $\hat{\mu }$ represents the weighted average of the variable of interest such as the different population groups (sex, age, rural, and urban, etc.). *h*_*i*_ represents the value of the variable of interest for individual *i*.

The difference in the concentration indexes between two periods was computed using the DASP menu in Stata (26), accounting for the full sampling design. This difference can result in a pro-poor “shift” or a pro-rich “shift” (see Table 2 for details). Briefly, a pro-poor “shift” occurs when the change (that is the difference) in the *CI*, between two time periods, is negative; while a pro-rich “shift” occurs if this change is positive. Table 2 summarizes the broad scenarios that can cause pro-poor and pro-rich “shifts” (27).

**Table 2:** Explaining a pro-poor and a pro-rich shift in the concentration index between two time periods.

**Pro-poor “shift”** Δ*CI* = *CI*_*t*_ − *CI*_*t*−1_ = Negative result	1. A previously pro-rich distribution becomes pro-poor (for example if *CI*_*t*−1_ = 0.5 and *CI*_*t*_ = −0.3 then Δ*CI* = −0.8) 2. A previously pro-poor distribution becomes more pro-poor (for example if *CI*_*t*−1_ = −0.5 and *CI*_*t*_ = −0.7 then Δ*CI* = −0.2) 3. A previously pro-rich distribution becomes less pro-rich (for example if *CI*_*t*−1_ = 0.5 and *CI*_*t*_ = 0.1 then Δ*CI* = −0.4)
**Pro-rich “shift”** Δ*CI* = *CI*_*t*_ − *CI*_*t*−1_ = Positive result	1. A previously pro-poor distribution becomes pro-rich(for example if *CI*_*t*−1_ = −0.3 and *CI*_*t*_ = 0.5 then Δ*CI* = 0.7) 2. A previously pro-poor distribution becomes less pro-poor(for example if (*CI*_*t*−1_ = −0.7 and *CI*_*t*_ = −0.5 then Δ*CI* = 0.2) 3. A previously pro-rich distribution becomes more pro-rich(for example if *CI*_*t*−1_ = 0.5 and *CI*_*t*_ = 0.7 then Δ*CI* = 0.2)

*Adapted from Ataguba (27)*.

*(1) Pro-rich and pro-poor, as used here, do not relate to any specific income thresholds of income or consumption. They are used to describe the direction of shifts in distributions either toward wealthier households (pro-rich) or poorer households (pro-poor)*.

*(2) For a previously pro-poor distribution, the original concentration index (CI_t−1_) is negative*.

*(3) For a previously pro-rich distribution, the original concentration index (CI_t−1_) is positive*.

The prevalence of alcohol consumption and *CI* estimates for all the surveys years are also reported. Only the 2008 and 2014/15 data are used to analyze the changes in socioeconomic inequalities. In fact, the results for shorter time periods (for example between 2008 and 2010) were not different from those presented in this paper.

## Results

### Prevalence of Current and Binge Drinkers by SES and Other Equity Stratifiers

As shown in Table 3, the proportion of current drinkers has increased over the years, from 26.9% in 2008 to 33.1% in 2014/15. African, Colored, all SES quintiles, adults aged 15–54 years old, rural and urban dwellers had experienced an increase in the prevalence of current drinking between 2008 and 2014/15. For the SES quintiles, the highest quintile has the highest current drinking rates, while the poorest has the lowest rates. The prevalence of current drinking decreased for the Asian/Indian, White and adults aged at least 55 years between 2008 and 2014/15.

**Table 3:** Prevalence of current alcohol drinkers in SA from 2008 to 2015 by SES and other equity stratifiers.

	**2008**	**2010/11**	**2012**	**2014/15**	**Total difference^**†**^**
Total	26.9%	26.2%	27.8%	33.1%	6.2%^***^
	(0.003)	(0.003)	(0.003)	(0.003)	(0.004)
Poorest	15.7%	15.2%	17.0%	23.1%	7.5%^***^
	(0.006)	(0.005)	(0.005)	(0.005)	(0.004)
2nd Quintile	21.3%	16.7%	19.8%	27.2%	5.9%^***^
	(0.006)	(0.006)	(0.006)	(0.006)	(0.004)
3rd Quintile	21.8%	25.0%	25.5%	32.1%	10.3%^***^
	(0.007)	(0.007)	(0.007)	(0.007)	(0.004)
4th Quintile	29.5%	27.6%	32.7%	36.7%	7.2%^***^
	(0.009)	(0.008)	(0.009)	(0.008)	(0.004)
Richest	45.8%	47.2%	44.3%	46.1%	0.3%
	(0.011)	(0.013)	(0.011)	(0.011)	(0.005)
*Chi-Square* (χ^2^)^a^	686.1^***^	361.2^***^	483.7^***^	342.3^***^	
Female	15.7%	14.4%	16.3%	20.2%	4.4%^***^
	(0.004)	(0.003)	(0.003)	(0.003)	(0.004)
Male	41.1%	40.2%	41.6%	47.7%	6.6%^***^
	(0.006)	(0.006)	(0.006)	(0.005)	(0.005)
*Chi-Square* (χ^2^)^a^	1.4e + 03^***^	1.5e + 03^***^	1.6e + 03^***^	2.2e + 03^***^	
African	21.5%	21.4%	23.5%	29.4%	7.9%^***^
	(0.004)	(0.003)	(0.003)	(0.003)	(0.004)
Colored	36.5%	34.8%	38.7%	45.2%	8.7%^***^
	(0.010)	(0.010)	(0.010)	(0.009)	(0.005)
Asian/Indian	32.3%	29.4%	26.7%	28.9%	−3.3%^***^
	(0.031)	(0.034)	(0.032)	(0.031)	(0.004)
White	58.3%	57.2%	54.0%	54.1%	−4.2%^***^
	(0.016)	(0.023)	(0.022)	(0.022)	(0.005)
*Chi-Square* (χ^2^)^a^	1.1e + 03^***^	443.9^***^	611.3^***^	557.1^***^	
15–24	19.7%	18.5%	19.6%	25.2%	5.5%^***^
	(0.006)	(0.005)	(0.005)	(0.005)	(0.004)
25–34	29.1%	30.2%	35.6%	42.5%	13.3%^***^
	(0.008)	(0.008)	(0.008)	(0.007)	(0.005)
35–44	32.0%	34.0%	32.3%	36.7%	4.7%^***^
	(0.009)	(0.009)	(0.009)	(0.008)	(0.004)
45–54	28.5%	29.5%	28.7%	35.0%	6.5%^***^
	(0.009)	(0.009)	(0.009)	(0.008)	(0.004)
55–64	32.5%	25.7%	26.6%	27.9%	−4.6%^***^
	(0.0124)	(0.011)	(0.010)	(0.010)	(0.004)
65+	25.9%	19.4%	20.7%	22.7%	−3.2%^***^
	(0.012)	(0.010)	(0.010)	(0.010)	(0.004)
*Chi-Square* (*χ^2^*)^a^	247.8^***^	272.8^***^	308.4^***^	580.6^***^	
Rural	17.4%	16.8%	19.5%	24.0%	6.6%^***^
	(0.004)	(0.004)	(0.004)	(0.004)	(0.004)
Urban	32.7%	32.2%	32.5%	38.1%	5.4%^***^
	(0.005)	(0.005)	(0.005)	(0.005)	(0.004)
*Chi-Square* (χ^2^)^a^	473.7^***^	417.6^***^	385.7^***^	553.1^***^	

† *Absolute difference between 2014/5 and 2008. Significance levels are denoted as follows:*

*** *p < 0.01*,

** *p < 0.05, and*

* *p < 0.10. Standard error displayed in parentheses*.

a *The Chi-Square (χ^2^) tests the relationship between current drinking and equity stratification variables as categorical variables*.

Binge drinkers (Table 4) increased slightly from 41.0% in 2008 to 43% in 2014/15. For the SES quintiles, the pattern is not uniform. The highest quintile has the lowest binge drinking rates; while the poorest and the fourth quintiles have very similar rates. Between 2008 and 2014/15, the proportion of binge drinkers increased in the poorest and the richest quintiles, while the middle three quintiles all had a decrease by 2014/2015 though there was much variability in the percentages over the time period. There was an increase in the prevalence of female binge drinking between 2008 and 2014/15, while the prevalence of male binge drinking remained approximately the same. Except for the African population group, the prevalence of binge drinking among the other race groups decreased between 2008 and 2014/15. Adults aged 25–34 and 55+ years experienced a decline in the prevalence of binge drinking, while those aged 15–24 years and 35–44 years had an increase in the prevalence of binge drinking. The prevalence of binge drinking declined in rural areas compared to a rise in urban areas between 2008 and 2014/5.

**Table 4:** Prevalence of binge drinkersª in SA from 2008 to 2015 by SES and other equity stratifiers.

	**2008**	**2010/11**	**2012**	**2014/15**	**Total Difference^**†**^**
Total	41.0%	41.0%	39.1%	43.0%	2.1%^**^
	(0.008)	(0.009)	(0.007)	(0.006)	(0.005)
Poorest	48.4%	45.4%	49.7%	49.6%	1.2%
	(0.021)	(0.020)	(0.018)	(0.014)	(0.005)
2nd Quintile	46.4%	52.9%	44.5%	44.1%	−2.3%^**^
	(0.017)	(0.019)	(0.016)	(0.012)	(0.005)
3rd Quintile	49.3%	48.5%	45.8%	46.2%	−3.0%^**^
	(0.018)	(0.018)	(0.016)	(0.013)	(0.005)
4th Quintile	51.0%	51.7%	41.2%	49.3%	−1.60%
	(0.018)	(0.019)	(0.017)	(0.014)	(0.005)
Richest	25.8%	25.4%	27.5%	31.9%	6.1%^***^
	(0.0149)	(0.020)	(0.016)	(0.016)	(0.005)
*Chi-Square* (*χ^2^*)^1^	148.0^***^	42.4^***^	38.78^***^	24.3^***^	
Female	24.8%	24.6%	25.8%	32.4%	7.6%^***^
	(0.012)	(0.014)	(0.012)	(0.010)	(0.004)
Male	48.8%	48.0%	45.3%	48.2%	−0.7%
	(0.010)	(0.010)	(0.009)	(0.008)	(0.005)
*Chi-Square* (*χ^2^*)^1^	125.9^***^	69.2^***^	94.0^***^	121.9^***^	
African	50.3%	52.2%	46.4%	50.4%	0.1%
	(0.010)	(0.010)	(0.009)	(0.007)	(0.005)
Colored	46.4%	41.0%	44.8%	43.9%	−2.4%^**^
	(0.018)	(0.020)	(0.016)	(0.014)	(0.005)
Asian/Indian	24.3%	8.8%	25.2%	22.7%	−1.5%^*^
	(0.058)	(0.042)	(0.065)	(0.059)	(0.004)
White	14.9%	12.3%	10.6%	11.6%	−3.3%^***^
	(0.015)	(0.023)	(0.018)	(0.019)	(0.003)
*Chi-Square* (*χ^2^*)^1^	277.0^***^	97.0^***^	151.9^***^	153.9^***^	
15–24	44.2%	44.2%	45.7%	47.9%	3.7%^***^
	(0.018)	(0.018)	(0.016)	(0.012)	(0.005)
25–34	51.4%	51.0%	45.4%	49.4%	−2.0%^*^
	(0.017)	(0.017)	(0.014)	(0.011)	(0.005)
35–44	41.3%	38.6%	39.8%	44.2%	2.9%^**^
	(0.018)	(0.020)	(0.018)	(0.015)	(0.005)
45–54	36.7%	37.1%	27.6%	38.7%	1.9%^*^
	(0.018)	(0.020)	(0.017)	(0.017)	(0.005)
55–64	30.4%	27.8%	32.7%	25.9%	−4.5%^***^
	(0.024)	(0.026)	(0.024)	(0.019)	(0.005)
65+	15.3%	17.8%	17.8%	14.8%	−0.5%
	(0.021)	(0.028)	(0.024)	(0.020)	(0.003)
*Chi-square* (*χ^2^*)^1^	100.7^***^	34.0^***^	104.6^***^	130.7^***^	
Rural	47.3%	51.4%	46.1%	44.0%	−3.3%^**^
	(0.014)	(0.014)	(0.012)	(0.010)	(0.005)
Urban	38.9%	37.7%	36.8%	42.7%	3.8%^***^
	(0.010)	(0.010)	(0.009)	(0.008)	(0.005)
*Chi-square* (*χ^2^*)^1^	5.0^***^	3.9^***^	7.5^***^	7.5^***^	

† *Absolute difference between 2014/5 and 2008. Significance levels are denoted as follows:*

*** *p < 0.01*,

** *p < 0.05, and*

* *p < 0.10. Standard errors displayed in parentheses*.

a *An adult (female or male) who is a current alcohol drinker consuming 5 or more standard drinks on a single occasion*.

1 *The Chi-Square (χ^2^) tests the relationship between binge drinking and equity stratification variables as categorical variables*.

### Assessing Socioeconomic Inequalities in Current and Binge Drinking

**Figure 1:**
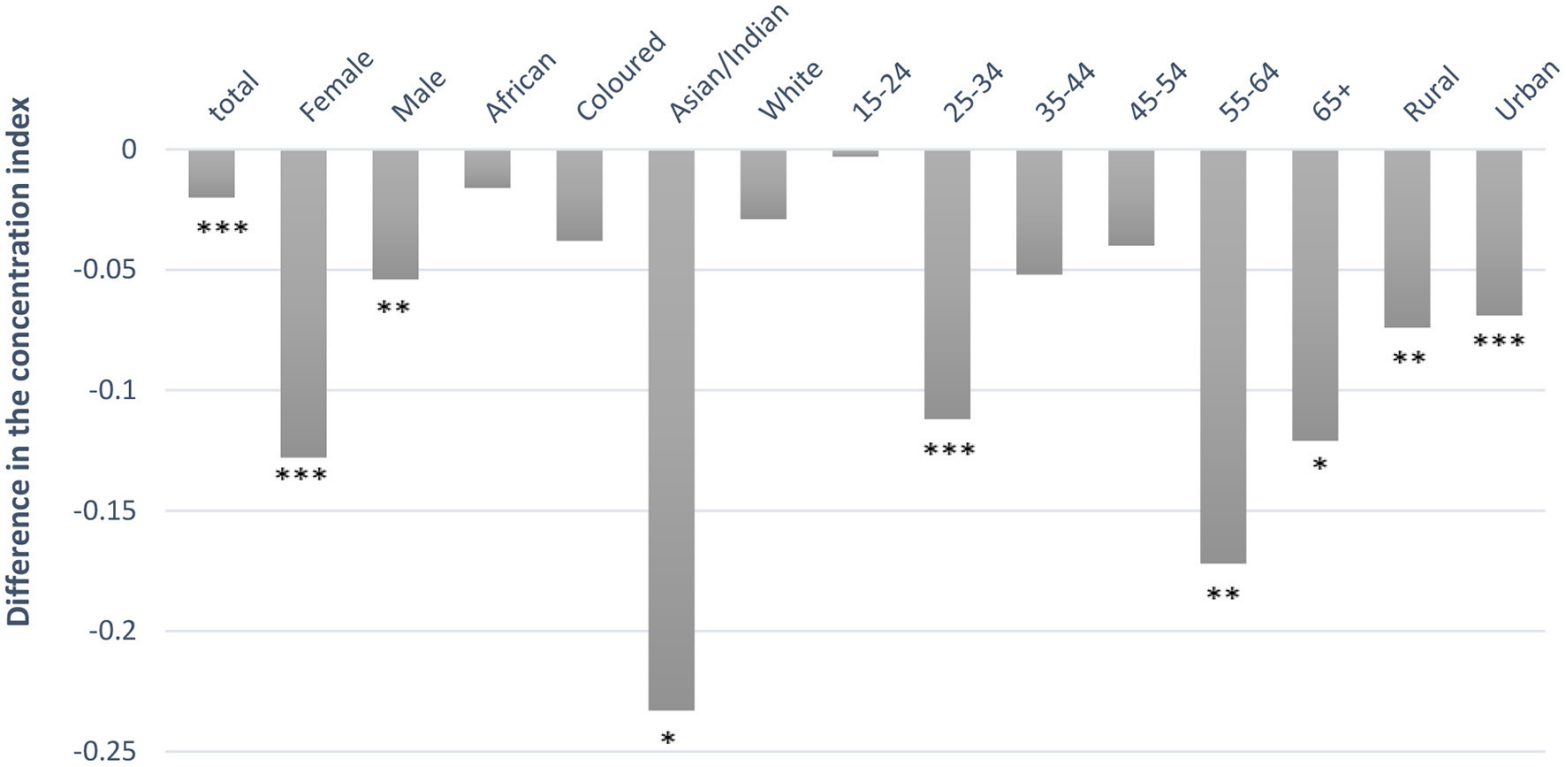
Difference in the concentration index of current drinkers between 2008 and 2014/5. A positive value signifies a pro-rich “shift” while a negative value signifies a pro-poor “shift.” Significance levels are denoted as follows: ***p < 0.01, **p < 0.05, and *p < 0.10.

**Figure 2:**
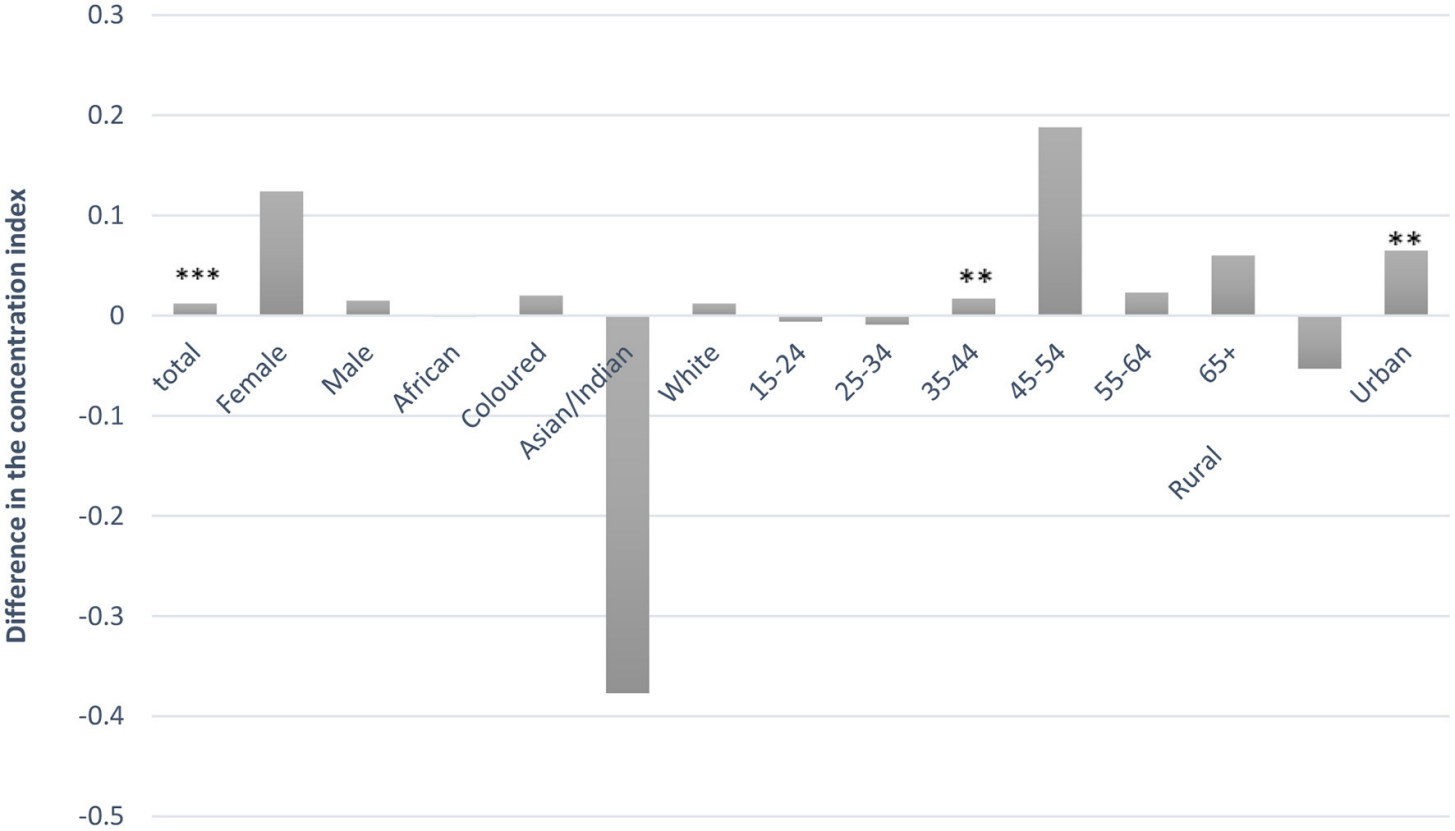
Difference in the concentration index of binge drinkers between 2008 and 2014/15. A positive value signifies a pro-rich “shift” while a negative value signifies a pro-poor “shift.” Significance levels are denoted as follows: ***p < 0.01, **p < 0.05, and *p < 0.10. ªAn adult (female or male) who is a current alcohol drinker consuming five or more standard drinks on a single occasion.

Table 5 shows that current drinking is more concentrated among richer individuals (a consequence of the positive concentration indexes in the “Total” row); while for binge drinking the pattern is not uniform. The concentration indexes for current drinkers remain positive while for binge drinkers, the concentration indexes changed over the years. The difference in the CIs between two time periods (2008 and 2014/15) indicates that current drinking, for all the equity stratifiers (sex, race, age, and rural, and urban), had a pro-poor “shift” (Figure 1). That is, the distribution of current drinkers was pro-rich in 2008 and became less pro-rich in 2014/15. Figure 2 shows that, besides the Asian/Indian and rural population, although not statistically significant, which had a pro-poor “shift,” binge drinking for all other equity stratifiers had a pro-rich “shift” between 2008 and 2014/15. That is, the distribution of overall binge drinkers was pro-poor in 2008 and became less pro-poor in 2014/15 (that is binge drinkers had shifted toward the richer group between 2008 and 2014/15).

**Table 5:** Concentration indexes of current drinkers and binge drinkers (2008–2015).

	**Current drinkers**				**Binge drinkers**^**a**^			
	**2008**	**2010/11**	**2012**	**2014/15**	**2008**	**2010/11**	**2012**	**2014/15**
Total^†^	0.047^***^	0.085^***^	0.083^***^	0.026^***^	−0.008	0.032	−0.031^*^	0.004
	(0.007)	(0.008)	(0.008)	(0.006)	(0.021)	(0.023)	(0.019)	(0.012)
Female	0.318^***^	0.345^***^	0.289^***^	0.190^***^	−0.298^***^	−0.325^***^	−0.236^***^	−0.174^***^
	(0.024)	(0.027)	(0.024)	(0.021)	(0.037)	(0.044)	(0.042)	(0.032)
Male	0.124^***^	0.153^***^	0.118^***^	0.069^***^	−0.065^***^	−0.079^**^	−0.097^***^	−0.050^**^
	(0.013)	(0.014)	(0.014)	(0.012)	(0.019)	(0.025)	(0.028)	(0.019)
**Race**								
African	0.136^***^	0.172^***^	0.171^***^	0.120^***^	0.037^**^	0.025	0.001	0.036^*^
	(0.015)	(0.015)	(0.014)	(0.011)	(0.017)	(0.018)	(0.019)	(0.013)
Colored	−0.001	0.045	0.029	−0.039	−0.087^*^	−0.001	−0.076	−0.0667^*^
	(0.035)	(0.043)	(0.032)	(0.025)	(0.047)	(0.080)	(0.046)	(0.035)
Asian/Indian	0.125	0.069	−0.070	−0.108	0.232	−0.153	−0.250	−0.145
	(0.089)	(0.103)	(0.110)	(0.094)	(0.179)	(0.221)	(0.170)	(0.196)
White	0.142^***^	0.185^***^	0.158^***^	0.113^**^	−0.117	0.010	0.160	−0.105
	(0.026)	(0.032)	(0.035)	(0.041)	(0.109)	(0.137)	(0.286)	(0.126)
**Age**								
15–24	0.193^***^	0.194^***^	0.208^***^	0.190^***^	−0.031	−0.076^**^	−0.064	−0.037
	(0.028)	(0.028)	(0.029)	(0.021)	(0.036)	(0.038)	(0.042)	(0.029)
25–34	0.188^***^	0.174^***^	0.180^***^	0.076^***^	−0.023^***^	−0.055^***^	−0.100^*^	−0.033
	(0.024)	(0.027)	(0.022)	(0.018)	(0.031)	(0.037)	(0.038)	(0.029)
35–44	0.163^***^	0.289^***^	0.169^***^	0.111^***^	−0.105^**^	−0.152^**^	−0.070	−0.088^**^
	(0.025)	(0.024)	(0.026)	(0.023)	(0.039)	(0.063)	(0.044)	(0.033)
45–54	0.189^***^	0.206^***^	0.189^***^	0.149^***^	−0.269^***^	−0.207^***^	−0.313^***^	−0.080
	(0.032)	(0.035)	(0.031)	(0.028)	(0.045)	(0.063)	(0.051)	(0.050)
55–64	0.274^***^	0.252^***^	0.229^***^	0.102^**^	−0.288^***^	−0.232^*^	−0.002	−0.265^***^
	(0.034)	(0.044)	(0.054)	(0.051)	(0.075)	(0.129)	(0.201)	(0.071)
65+	0.354^***^	0.341^***^	0.259^***^	0.233^***^	−0.497	−0.410^***^	−0.522^***^	−0.436^***^
	(0.033)	(0.055)	(0.051)	(0.052)	(0.068)	(0.096)	(0.065)	(0.075)
**Rural/Urban**								
Rural	0.192	0.188^***^	0.159^***^	0.119^***^	0.045^*^	0.026	−0.005	−0.008
	(0.021)	(0.020)	(0.020)	(0.016)	(0.026)	(0.023)	(0.025)	(0.021)
Urban	0.165^***^	0.197^***^	0.169^***^	0.096^***^	−0.181^***^	−0.173^***^	−0.166^***^	−0.116^***^
	(0.015)	(0.017)	(0.016)	(0.013)	(0.023)	(0.031)	(0.034)	(0.021)

*Significance levels are denoted as follows*:

*** *p < 0.01*,

** *p < 0.05, and*

* *p < 0.10. Standard error displayed in parentheses*.

a *An adult (female or male) who is a current alcohol drinker consuming five or more standard drinks on a single occasion*.

† *Convenient regression controlling for gender, race, age, and urban was used to calculate the concentration index*.

## Discussion

The overall results show that in SA, the prevalence of alcohol use and binge drinking increased between 2008 and 2015. Besides, the Asian/Indian and Colored population, where current drinkers are concentrated more among the low-SES groups, current drinkers for all other equity stratifiers remain concentrated among the wealthier SES group; whereas binge drinkers (assessed among current drinkers) are concentrated among the poorer SES group.

Results emerging from the analysis of the concentration indexes are consistent with inequalities in alcohol consumption patterns seen in the literature (28–30). For instance, Wood and Bellis (28) assessed socioeconomic inequality in alcohol consumption in European countries. Similar to our results, Wood and Bellis (28) found that, overall, individuals with high socioeconomic status (for males and females) are more likely to be current drinkers. Binge drinkers were found to be more concentrated among adults (males and females) with low SES; however, not all European countries had the same pattern. For instance, Portugal and Hungary reported binge drinking concentrated among the richest for males; while in Germany, binge drinking was more prevalent among the richest for females. Combes et al. (29) analyzed income inequality in alcohol consumption in Sweden over 8 years and found that inequality in alcohol consumption is pro-rich. While in Germany, Pabst et al. (31) found a positive association between SES and alcohol consumption. The study by Lawana and Booysen (17), the only South African study looking at socioeconomic inequality in alcohol consumption in informal settlements in SA, demonstrated results differing from those reported elsewhere in the literature. Their study found that men living in an informal settlement have a pro-poor alcohol consumption distribution. As mentioned in their research, one of the reasons why Lawana and Booysen's (17) results differ from those in the literature may be due to the use of a wealth index instead of income to measure socioeconomic inequality and the possibility of limited range of SES across which to measure distribution since their study targeted men living in a low socioeconomic area.

This paper found that socioeconomic inequality in alcohol consumption for current drinkers had a pro-poor “shift”; while binge drinkers had a pro-rich “shift” between 2008 and 2014/15. Current drinkers were more concentrated among the richer adults in 2008, but this concentration among richer SES groups decreased in 2014/15. Binge drinkers, on the other hand, are more concentrated among poorer adults, but this concentration among the poorer SES group decreased between 2008 and 2014/15. This result was the case irrespective of gender, signifying that the prevalence of both current and binge drinking between wealthier and the poorer SES groups is slowly converging.

A possible explanation for the pro-poor “shift” in current drinkers found in this paper could be an increase in overall alcohol consumption in SA, especially among low-SES adults. For both sexes, the concentration indexes were positive (pro-rich) for current drinking in all years, while the concentration indexes for binge drinking in 2008 changed over the years. When considering race profiles, among Africans, current drinkers and binge drinkers were concentrated among wealthier SES groups. This shift in alcohol consumption toward richer adults might be explained by the phenomenon of a recent growing African middle class (32).

The temporal increase in current drinkers and binge drinkers found in this paper is not surprising. Research suggests that one of the factors responsible for increases in alcohol consumption in Africa is the aggressive marketing strategies adopted by the alcohol industry (33–35). For instance, alcohol industries create new products such as the ready to drink beverages (RTDs) to attract new consumers (especially young people and women). Among other things, they are promoting drinking as a tradition and part of the culture, sponsoring sports events and celebrities to create the image that drinking alcohol is “cool” and suggesting alcohol is “good for health” (33). It seems that these marketing strategies have been successful in increasing consumption levels. According to the South African Wine Industry Information and System (SAWIS) data, all alcohol volume in the country increased by 12.3% (3.5–3.9 billion liters) between 2005/06 and 2014/15 (36).

The total alcohol per capita consumption in SA is expected to increase from 11.5 L of pure alcohol in 2015 to 12.1 in 2025 (5). Therefore, unless there are major public health and policy interventions, alcohol-related harms are likely to increase due to increased exposure to alcohol consumption (for example drinking high level of alcohol in one occasion) (1, 33). Results in this paper identify the sociodemographic groups that are more likely to engage in high-risk drinking and be exposed to alcohol-related harms. Thus, to reduce risk by decreasing consumption levels, especially harmful consumption, this paper emphasizes that policies should target both the factors that increase people's susceptibility to the consequences of alcohol use [for example access to alcohol retail outlets and alcohol advertising (37, 38)], as well as measures to reduce or mitigate rising alcohol consumption [for example alcohol excise taxes (16, 39–45)].

### Limitation and Strength of This Study

One of the study's strengths is that socioeconomic inequality in alcohol consumption was assessed using comparable nationally representative data that span more than 5 years. Also, this analysis provides an initial attempt to assess socioeconomic disparity in alcohol consumption over time using the concentration index that is suitable for assessing socioeconomic inequality. A study limitation is that the same amount of standard drinking was used to generate the binge drinking and current drinking variables for males and females. Although the international literature suggests the use of 4 standard drinks as the benchmark for females, this was not possible as the NIDS dataset uses the same criterion for males and females. In addition, the NIDS adults' questionnaire does not specify the timeframe of consumption. This study also potentially underestimates socioeconomic inequality in alcohol consumption due to the underreporting of consumption often noted in the literature such as stigma, poor memory, bias, wording and timeframes of the survey questions, differences in standard drink according to the type of beverage preference by SES, informal brewing and consumption of alcohol, amongst other reasons (4, 46). For instance, women and individuals with religious affiliation might be less likely to report binge drinking. However, the under-reporting might have a small impact on the results of equity analyses since the underreporting of consumption may differ by level of alcohol risk drinking rather than SES (47, 48).

Also, differences in standard drinking according to the type of beverage preference by SES (15, 16) may result in differences in the total quantity of standard drinking by SES. However, the bias might have a small impact on the results with similar patterns in underreporting of consumption by SES. Another limitation is the exclusion of the latest NIDS (2017-Wave 5) data. As noted earlier, alcohol questions were dropped from the wave 5 survey as the NIDs team could not get ethics approval (for fielding the alcohol questions) in time for the fieldwork.

## Conclusion

This paper provides insight into the prevalence of alcohol consumption by demographic subgroups in SA. It also provides detailed insights into the magnitude and changing patterns of the socioeconomic inequality in alcohol consumption in the country. The results show that the pattern of socioeconomic disparity in alcohol consumption in SA varied across demographic groups and had changed over time. Also, the results show that binge drinking is a bigger problem among selected population groups such the low-SES, young individuals, male and African populations. Based on the results, the SA government should continue to push forward policies aiming to decrease alcohol consumption for those who are more exposed and vulnerable to alcohol harms. For instance, the SA government should reduce access to retail outlets, especially in deprived neighborhoods. In addition, shebeens, an informal unlicensed drinking place in a township, should be regularized to control the quality and quantity of alcohol sold. To decrease young individuals' consumptions, SA government should increase age restrictions on the sale of alcohol (from 18 to 21 years) and ban alcohol advertising close to schools and in sports—from its junior competitions and at the national level.

## Data Availability Statement

The original contributions presented in the study are included in the article/supplementary material, further inquiries can be directed to the corresponding author/s.

## Ethics Statement

The study was approved by the Human Research Ethics Committee of the Faculty of Health Sciences at the University of Cape Town (HREC reference number: 798/2017).

## Author Contributions

MF carried out the statistical analysis and led the writing of the manuscript. All authors helped to conceptualize the research, reviewed the results, helped to revise the manuscript, and approved the manuscript submitted for publication.

## Conflict of Interest

The authors declare that the research was conducted in the absence of any commercial or financial relationships that could be construed as a potential conflict of interest.

## Abbreviations

DALYs: Disability-adjusted life years lost
CPI: Consumer Price Index
SES: Socioeconomic status
NIDS: National Income Dynamics Study
CSMs: Continuing sample members
SALDRU: The Southern Africa Labour and Development Research Unit
DASP: Distributive Analysis Stata Package
CI: Concentration index
SAWIS: South African Wine Industry Information and System
RTDs: Ready to drink beverages.
